# Supplementary material for: Care for post-COVID-19 condition in Germany from the perspectives of patients, informal caregivers and general practitioners: Study protocol for a mixed methods study
Source: PLoS One. 2024 Dec 31;19(12):e0316335. doi: 10.1371/journal.pone.0316335 (PMC11687889; doi:10.1371/journal.pone.0316335)
Supplement: S1 Appendix — (PDF) [file pone.0316335.s001.pdf]

S1 Appendix: Self-developed items included in both online surveys of affected individuals

General questions about the disease/infection:

Question category	Answer options
Actuality of PCC	yes<br>no, not anymore<br>no, never
Begin/End PCC (year and month)	dropdown
Time of SARS-CoV-2-infection (year and month)	dropdown
Course of SARS-CoV-2-infection	no symptoms<br>light symptoms<br>moderately difficult<br>severe symptoms
Medical institution used for SARS-CoV-2-infection	no use<br>general practitioner<br>specialist<br>outpatient care in hospital<br>inpatient care in hospital<br>intensive care unit
Number of SARS-CoV-2-infections	numeric input
Number of vaccinations against COVID-19	no vaccination<br>yes, once<br>yes, twice<br>yes, more than twice<br>no answer
Number of vaccinations against COVID-19 before begin of PCC	no vaccination<br>yes, once<br>yes, twice<br>yes, more than twice<br>don’t know

PCC-symptoms:

Question category	Answer options
PCC-symptoms	14 different symptoms[1]<br>currently present<br>present for a while<br>never present
Impairment due to PCC (currently)	14 different symptoms[1]<br>very strong<br>strong<br>moderate<br>light<br>no impairment
Impairment due PCC at worst time	14 different symptoms[1]<br>very strong<br>strong<br>moderate<br>light<br>no

Improvement of PCC-symptoms	yes<br>no
Believe in disappearance of symptoms	yes<br>no

[1]**Post-Covid-symptoms:**

- fatigue
- shortness of breath
- brain fog
- difficulty concentrating
- loss of smell/taste
- chest pain
- cough
- headache
- memory symptoms
- joint pain
- muscle pain/cramps
- long recovery phase after light exertion
- sleep disorder
- heart palpitation

Utilisation of medical services:

**Question category**	**Answer options**
Inpatient admission	yes<br>no
Time of inpatient stay	numeric input
Utilisation of rehabilitation for PCC	yes, outpatient<br>yes, inpatient<br>no
Time of rehabilitation	numeric input
Doctor’s visits	8 different specialities [2]<br>yes<br>no
Number of doctor’s visits	8 different specialities [2]<br>numeric input
Time required for doctor’s visit	8 different specialities [2]<br>numeric input
Satisfaction with outpatient care	8 different specialities [2]<br>very unsatisfied<br>unsatisfied<br>partly satisfied/unsatisfied<br>satisfied<br>very satisfied
Feel taken seriously by doctors	8 different specialities [2]<br>yes<br>rather yes<br>partly yes/partly no<br>rather no<br>no

Therapies utilised	23 different therapies[3] and free text<br>yes<br>no
Frequency of therapies utilised	23 different therapies[3] and free text<br>numeric input
Time required for therapy utilisation	23 different therapies[3] and free text<br>numeric input
Effect of utilised therapies	23 different therapies[3] and free text<br>significantly improved<br>partly improved<br>neither improved nor worsened<br>partly worsened<br>significantly worsened
Private payment for therapies	23 different therapies[3] and free text<br>complete<br>partly<br>no
Reasons for not taking up therapies	23 different therapies[3] and free text<br>don’t know it<br>no medical recommendation<br>no need/ no<br>no appointment<br>not in reachable distance<br>to expensive<br>other reasons
Medicaments/medicine	pain reliever<br>homeopathic<br>dietary supplements<br>others<br>free text
Satisfaction with healthcare	very unsatisfied<br>unsatisfied<br>partly satisfied/unsatisfied<br>satisfied<br>very satisfied

[2]Specialities:

- general practitioner
- neurologist
- pulmonologist
- cardiologist
- internist
- post-covid outpatient clinic
- psychiatrist, psychosomaticist or psychotherapist
- alternative practitioner

[3]Therapies:

- physiotherapy/physical therapy
- massage
- respiratory therapy
- speech therapy
- osteopathy

- occupational therapy
- relaxation therapy
- psychological counseling
- psychotherapy
- rehabilitation sport
- functional training/sports therapy
- acupuncture
- traditional Chinese medicine
- repetitive transcranial magnetic stimulation
- ketamine therapy
- ozone therapy
- hormone analysis
- blood dialysis
- colon irrigation
- oxygen chamber
- cold chamber
- homeopathy
- hypnosis

Work life:

Question category	Answer options
Work status	unemployed<br>employed
PCC is reason for unemployment	yes<br>no<br>not fitting (student, pensioner)
Unemployed due to PCC	yes<br>no
Reduction in working hours due to PCC	yes<br>no
Job change due to PCC	yes<br>no
Days of incapacity for work due to PCC	numeric input
Impairment at work due to PCC	yes<br>no
Satisfaction with job	very unsatisfied<br>unsatisfied<br>partly satisfied/unsatisfied<br>satisfied<br>very satisfied

Daily life:

Question category	Answer options
Frequency of restriction due to PCC in daily life	(almost) daily<br>several times a week<br>once a week<br>several times a month<br>once a month<br>(almost) never

Number of informal caregivers	numeric input
Frequency and duration of help from informal caregivers	numeric input
Feeling of being a burden on informal caregivers	yes<br>rather yes<br>partly/partly<br>rather no<br>no
Feel taken seriously by informal caregivers	yes<br>rather yes<br>partly/partly<br>rather no<br>no
Known persons with PCC	yes, one known person<br>yes, several known persons<br>no
Membership in self-help group	yes, rather active member<br>yes, rather passive member<br>no

Chronic disease:

**Question category**	**Answer options**
Chronic disease existent before PCC	yes<br>no
Type of chronic disease	yes or no<br>asthma<br>chronic lung disease<br>heart disease<br>high blood pressure/hypertension<br>chronic complaints after a stroke<br>chronic complaints in the lower back/lumbar spine<br>chronic complaints in the neck/cervical spine<br>osteoarthritis<br>diabetes<br>allergies, non-allergic asthma<br>liver cirrhosis<br>chronic kidney problems or kidney failure<br>elevated blood lipids or cholesterol levels<br>depression<br>anxiety disorder<br>cancer<br>epilepsy<br>multiple sclerosis<br>rheumatoid arthritis<br>other

Sociodemographic and socioeconomic factors:

Question category	Answer options
Sex	male<br>female<br>diverse
Age	18-25<br>groups of 5 years each<br>> 80
School leaving certificate	7 different German school leaving certification possibilities and free text
Graduation degree	8 different German graduation degree possibilities and free text
German as first language	yes<br>no
German skills	poor<br>little<br>fair Good<br>very good
Housing area	metropolis<br>suburb of a large city<br>medium-sized or small town<br>rural village
Partnership	yes<br>no
Persons in household	numeric input
Children under 14 years in household	numeric input
Monthly net household income	under 1.000 €<br>steps of 500 € each<br>over 6.000 €
Comments	free text
