# Supplementary material for: Care for post-COVID-19 condition in Germany from the perspectives of patients, informal caregivers and general practitioners: Study protocol for a mixed methods study
Source: PLoS One. 2024 Dec 31;19(12):e0316335. doi: 10.1371/journal.pone.0316335 (PMC11687889; doi:10.1371/journal.pone.0316335)
Supplement: S2 Appendix — (PDF) [file pone.0316335.s002.pdf]

S2 Appendix: Self-developed items included in the online survey of informal caregivers

General question:

Question category	Answer options
Current support for person with PCC	yes<br>no, as the person no longer needs support<br>no (other reasons)

Need for support:

Question category	Answer options
Relationship to the person with PCC	partner<br>parent/child<br>other family member<br>friend<br>neighbor<br>acquaintance<br>colleague
Frequency of support for the person with PCC	7 different tasks[1] and free text<br>(Almost) daily<br>several times a week<br>once a week<br>several times a month<br>once a month<br>(Almost) never
Support time	numeric input

[1]tasks:

- household
- shopping
- childcare
- personal hygiene
- driving service
- accompaniment to doctor's appointment
- emotional support/conversations

(Emotional) stress:

Question category	Answer options
Change in need for support over time	yes, symptoms improved<br>yes, person has adapted<br>no
Believe in disappearance of symptoms	yes<br>no

Sociodemographic facts:

Question category	Answer options
Sex	male<br>female<br>diverse
Age	18-25<br>groups of 5 years each<br>> 80
School leaving certificate	7 different german school leaving certification possibilities and free text
Graduation degree	8 different german graduation degree possibilities and free text
German as first language	yes<br>no
German skills	poor<br>little<br>fair good<br>very good
Housing area	metropolis<br>outskirts or suburb of a large city<br>medium-sized or small town<br>rural village
Partnership	yes<br>no
Persons in household	numeric input
Children under 14 years in household	numeric input
Monthly net household income	under 1.000 €<br>in steps of 500 € each<br>over 6.000 €
Comments	free text
