# Supplementary material for: Care for post-COVID-19 condition in Germany from the perspectives of patients, informal caregivers and general practitioners: Study protocol for a mixed methods study
Source: PLoS One. 2024 Dec 31;19(12):e0316335. doi: 10.1371/journal.pone.0316335 (PMC11687889; doi:10.1371/journal.pone.0316335)
Supplement: S3 Appendix — (PDF) [file pone.0316335.s003.pdf]

S3 Appendix: Guidelines for semi-structured interviews with PCC patients and their informal caregivers

**Topic 1: Experiences with post-COVID symptoms**	
**Key question**	**In-depth inquiries**
Please tell me about the **symptoms** that you associate with your post-COVID condition.	• Which symptoms do/did you perceive as particularly stressful and which as less stressful?<br>• How do/did your symptoms affect your mood?<br>• How do/did your symptoms affect your everyday life?
**Topic 2: Experiences with the post-COVID condition in the social environment**	
**Opening question**	
Please tell me about your **living conditions** – Where and how do you live? And with which people do you spend your daily life?	
**Key question**	**In-depth inquiries**
Please tell me how people in your **private environment** (e.g. partner, family, friends, neighbors) deal or have dealt with your post-COVID symptoms.	• How do people around you perceive your post-COVID condition and how do they react to it?<br>• How do these reactions affect you, how do they make you feel?<br>• To what extent have your relationships with other people changed since your post-COVID condition?<br>• What impact does your post-COVID condition have on your relatives? To what extent do you have the impression that your relatives feel burdened by it?<br>• What ways and possible solutions have you and the people around you found to cope with your post-COVID related limitations and better manage your symptoms? What worked, what did not work?<br>• To what extent were tasks redistributed, daily routines adapted or changed? How did these changes make you feel?<br>• To what extent do or did you and your relatives feel that you have to "reinvent" yourself or develop new goals in life due to your post-COVID condition?

<table>
<tr><th colspan="2">Topic 3: Experiences with the post-COVID condition at work</th></tr>
<tr><td colspan="2">Opening question</td></tr>
<tr><td colspan="2">Please tell me about your work and what your daily working life looks like.</td></tr>
<tr><td>Key question</td><td>In-depth inquiries</td></tr>
<tr><td rowspan="2">Please tell me how your post-COVID symptoms affect or have affected your employment.</td><td>The interviewee is or was employed while experiencing post-COVID symptoms:
• How do/did people at your work perceive or react to your post-COVID condition?
• How does/did this affect you and how does/did it make you feel?
• How do/did you, your colleagues, superiors and/or employees deal with your post-COVID related limitations at work?
• What ways and possible solutions have you, your colleagues, superiors and/or employees found to cope better with your limitations and symptoms?
• To what extent has your relationship with your colleagues, superiors and/or employees changed?</td></tr>
<tr><td>The interviewee is currently not employed due to post-COVID:
• What are your current thoughts and feelings about work? Can you imagine returning to work again?</td></tr>
<tr><th colspan="2">Topic 4: Experiences in the healthcare system with the post-COVID condition</th></tr>
<tr><td>Please tell me about your experiences in the healthcare system in relation to your post-COVID condition.</td><td>• Which doctors have you seen with your post-COVID symptoms and what examinations and treatments have you received?
• Please describe how you experience or have experienced the role of your family doctor in this context.
• Please tell me how you experience or have experienced the role of medical specialists in this context.
• What was the process leading up to the diagnosis?
• What happened after your post-COVID condition was diagnosed? Where were you referred to and what were the different stations in your patient journey? (e.g. medical specialists, rehabilitation, hospital, physiotherapy, counseling, psychotherapy)</td></tr>
</table>

	• How did people in the healthcare system react to you and your post-COVID symptoms and how did these reactions make you feel?<br>• How do you deal with the uncertainties associated with a very sparsely researched disease? How do other people deal with it?<br>• We are also interested in alternative treatment methods, e.g. homeopathy, singing bowl therapy, or acupuncture. What experiences, if any, have you had with these?<br>• What experiences have you had with psychological support services, e.g. counseling or therapy?<br>• How easy or difficult did you find it to get support and information for your post-COVID symptoms?<br>• What did you feel was missing in the provided healthcare services and what would you like to see in the future for the care of post-COVID patients?
**Topic 5: Experiences of relatives dealing with the post-COVID condition**	
**Key question**	**In-depth inquiries**
You have now listened to the conversation with your XXX. How do you deal with your XXX's **post-COVID condition**? Please tell me about it.	• What is your relationship like with your XXX and how has it changed as a result of the post-COVID condition?<br>• To what extent do you feel burdened by your XXX's post-COVID condition?<br>• What kind of support would you like to see for relatives of post-COVID patients?
**Final questions**	
• Well, now we have talked about quite a few things. Is there anything else you would like to add that is important to you and that has not yet been discussed in this interview?<br>• Can you briefly tell me about your motivation to take part in this interview?	
