# Supplementary material for: Care for post-COVID-19 condition in Germany from the perspectives of patients, informal caregivers and general practitioners: Study protocol for a mixed methods study
Source: PLoS One. 2024 Dec 31;19(12):e0316335. doi: 10.1371/journal.pone.0316335 (PMC11687889; doi:10.1371/journal.pone.0316335)
Supplement: S1 Checklist — (PDF) [file pone.0316335.s004.pdf]

STROBE Statement—checklist of items that should be included in reports of observational studies

	Item No.	Recommendation	Page No.	Relevant text from manuscript
**Title and abstract**	1	(*a*) Indicate the study's design with a commonly used term in the title or the abstract	p. 1	"Care for post-COVID-19 condition in Germany from the perspectives of patients, informal caregivers and general practitioners: Study protocol for a mixed methods study"
		(*b*) Provide in the abstract an informative and balanced summary of what was done and what was found	p. 3	*See methods section in abstract.*
**Introduction**				
Background/rationale	2	Explain the scientific background and rationale for the investigation being reported	p. 5-6	*See introduction.*
Objectives	3	State specific objectives, including any prespecified hypotheses	p. 7-8	*See objectives.*
**Methods**				
Study design	4	Present key elements of study design early in the paper	p. 8; Figure 1	"The study employs a mixed methods design, integrating quantitative and qualitative methods of health services research, secondary data analysis and health economic analysis (Fig 1)."
Setting	5	Describe the setting, locations, and relevant dates, including periods of recruitment, exposure, follow-up, and data collection	p. 8-14	*See data collection.*
Participants	6	(*a*) *Cohort study*—Give the eligibility criteria, and the sources and methods of selection of participants. Describe methods of follow-up<br>*Case-control study*—Give the eligibility criteria, and the sources and methods of case ascertainment and control selection. Give the rationale for the choice of cases and controls<br>*Cross-sectional study*—Give the eligibility criteria, and the sources and methods of selection of participants	p.9	Affected and formerly affected individuals:<br>Group 1/3: "[…] adult individuals meeting the following inclusion criteria are invited by post to participate in the research: (a) insured with AOK Lower Saxony, (b) diagnosed with PCC (ICD10 U09.9) in their outpatient billing data or a certificate of incapacity for work in 2022, (c) resident of Lower Saxony and (d) continuously insured with AOK Lower Saxony since 2019. Individuals whose affairs are managed by a guardian or who are employees of the health insurance company are excluded"

			p.10	Group 2: "Eligible participants meet the following criteria: (a) tested positive for SARS-CoV-2 in 2023 (self-test sufficient), (b) report persistent symptoms and (c) reside in Lower Saxony (group 2)."<br><br>Informal caregivers:
			p.13	"Eligible informal caregivers must be ≥ 18 years old and the primary confidant of the affected or formerly affected individual, and they must provide care or emotional support to them."<br><br>General practitioners:
			p.14	"[…] all registered and practicing GPs in Lower Saxony, except those treating only privately insured patients, are eligible"
		(*b*) *Cohort study*—For matched studies, give matching criteria and number of exposed and unexposed<br>*Case-control study*—For matched studies, give matching criteria and the number of controls per case	n/a	
Variables	7	Clearly define all outcomes, exposures, predictors, potential confounders, and effect modifiers. Give diagnostic criteria, if applicable	n/a	
Data sources/ measurement	8*	For each variable of interest, give sources of data and details of methods of assessment (measurement). Describe comparability of assessment methods if there is more than one group	p. 8-14	*See data collection.*
Bias	9	Describe any efforts to address potential sources of bias	n/a	
Study size	10	Explain how the study size was arrived at	n/a	

Continued on next page

Quantitative variables	11	Explain how quantitative variables were handled in the analyses. If applicable, describe which groupings were chosen and why	p. 4	*See Data analysis.*
Statistical methods	12	(*a*) Describe all statistical methods, including those used to control for confounding	p. 13	*See Data analysis.*
		(*b*) Describe any methods used to examine subgroups and interactions	p. 14-15	"First, bivariate statistics will be used to analyse the patient data, with the aim of determining associations between patient-related constructs and outcomes and utilisation of medical services. In addition, the association between PCC symptom severity and informal caregiver burden will be determined. Second, multivariable regression models will be applied to deter-mine relevant influencing factors for the utilisation of medical services.."
		(*c*) Explain how missing data were addressed	n/a	
		(*d*) *Cohort study*—If applicable, explain how loss to follow-up was addressed<br>*Case-control study*—If applicable, explain how matching of cases and controls was addressed<br>*Cross-sectional study*—If applicable, describe analytical methods taking account of sampling strategy	n/a	
		(*e*) Describe any sensitivity analyses	n/a	
**Results**				
Participants	13*	(a) Report numbers of individuals at each stage of study—eg numbers potentially eligible, examined for eligibility, confirmed eligible, included in the study, completing follow-up, and analysed	n/a	
		(b) Give reasons for non-participation at each stage	n/a	
		(c) Consider use of a flow diagram	n/a	
Descriptive data	14*	(a) Give characteristics of study participants (eg demographic, clinical, social) and information on exposures and potential confounders	n/a	
		(b) Indicate number of participants with missing data for each variable of interest	n/a	

		(c) *Cohort study*—Summarise follow-up time (eg, average and total amount)	n/a
Outcome data	15*	*Cohort study*—Report numbers of outcome events or summary measures over time	n/a
		Case-control study—Report numbers in each exposure category, or summary measures of exposure	n/a
		Cross-sectional study—Report numbers of outcome events or summary measures	n/a
Main results	16	(*a*) Give unadjusted estimates and, if applicable, confounder-adjusted estimates and their precision (eg, 95% confidence interval). Make clear which confounders were adjusted for and why they were included	n/a
		(*b*) Report category boundaries when continuous variables were categorized	n/a
		(*c*) If relevant, consider translating estimates of relative risk into absolute risk for a meaningful time period	n/a

Continued on next page

Other analyses	17	Report other analyses done—eg analyses of subgroups and interactions, and sensitivity analyses	n/a	
**Discussion**				
Key results	18	Summarise key results with reference to study objectives	n/a	
Limitations	19	Discuss limitations of the study, taking into account sources of potential bias or imprecision. Discuss both direction and magnitude of any potential bias	p.16-17	See discussion.
Interpretation	20	Give a cautious overall interpretation of results considering objectives, limitations, multiplicity of analyses, results from similar studies, and other relevant evidence	n/a	
Generalisability	21	Discuss the generalisability (external validity) of the study results	n/a	
**Other information**				
Funding	22	Give the source of funding and the role of the funders for the present study and, if applicable, for the original study on which the present article is based	p. 25	"The present study is supported by the COVID-19-Research Network Lower Saxony (COFONI), through funding from the Ministry of Science and Culture of Lower Saxony in Germany (14-76403-184)."

*Give information separately for cases and controls in case-control studies and, if applicable, for exposed and unexposed groups in cohort and cross-sectional studies.

Note: An Explanation and Elaboration article discusses each checklist item and gives methodological background and published examples of transparent reporting. The STROBE checklist is best used in conjunction with this article (freely available on the Web sites of PLoS Medicine at http://www.plosmedicine.org/, Annals of Internal Medicine at http://www.annals.org/, and Epidemiology at http://www.epidem.com/). Information on the STROBE Initiative is available at www.strobe-statement.org.
