# Supplementary material for: Care for post-COVID-19 condition in Germany from the perspectives of patients, informal caregivers and general practitioners: Study protocol for a mixed methods study
Source: PLoS One. 2024 Dec 31;19(12):e0316335. doi: 10.1371/journal.pone.0316335 (PMC11687889; doi:10.1371/journal.pone.0316335)
Supplement: Attachment — Submitted filename: Response to Reviewers_2.pdf [file pone.0316335.s006.pdf]

Dear editor,

We would like to thank reviewer 1 for the positive review and reviewer 2 for the useful comments. We feel that the comments of reviewer 2 refer to the manuscript originally submitted in March rather than the revised version submitted in September, as almost all of the comments have already been addressed in the revised version on the basis of very similar comments from reviewer 1. Nevertheless, we have taken the helpful suggestions of reviewer 2 into account and made an addition in lines 244-248.

Please find below our answers to the comments of the reviewer 2.

Kind regards,

Maike Stolz, on behalf of all authors.

Reviewer comments

Reviewer #1: Thank you for considering my remarks. I feel that have been adequately addressed.
i have no further comments.

Thank you for the positive review.

Reviewer #2: The study is interesting and the use of mixed method to reduce confounders and biases makes the protocol original. However, there are some issues that should be in addressed:

Thank you very much for your comprehensive review and helpful suggestions.

We feel that your comments refer to the manuscript originally submitted in March rather than the revised version submitted in September, as almost all of your comments have already been addressed in this version on the basis of very similar comments from a second reviewer.

Please find the corresponding changes below.

1. Define informal health care givers and the scope of the GPs to be included in the survey.

We included the definition of informal caregivers in the introduction.

Line 92-95: "The functional limitations associated with PCC impact patients' social lives and fulfilment of everyday tasks [3, 4], thereby necessitating the support services of informal caregivers (non-professional persons (both kin and non-kin caregivers like neighbours and friends) who provide care in a home setting)."

We are not quite sure what is meant by 'scope of the GPs included'. To be invited to the survey, the only requirement is that you are registered as a GP with a license from the statutory health insurance provider and are actively practicing. To participate in the preparatory focus groups, the GPs must also have treated patients with PCC. We have added this accordingly.

Line 310-311: GPs who treat or have treated people with PCC and are interested in taking part will be invited to contact the research team by telephone or email.

2. When referring PCC in the objectives section use the formal definition used in the introduction section of the protocol. Why FS, as it has different ICD and will confound the results.

Thank you for pointing this out. We will now not put a special emphasis on "fatigue syndrome", but mention fatigue (measured on the Chalder Fatigue Scale) as one of the most common symptoms of PCC. We have changed the entire manuscript accordingly, especially in the "Objectives" section (line 134-152).

3. Why are the second survey group selected as self-selection, as this will lead to bias. What other strategies did you use to reduce bias other than the DSQ-PEM.

The self-selected sample was integrated in order to reach people who had not yet seen a GP or specialist or had not received a PCC diagnosis. This was done on the assumption that this group could differ from the AOK sample, e.g. with regard to the utilisation of alternative medical measures which are not included in the claims data. There are some additional questions asked to validate the self-report. We added this information in the methods section.

Line 219-224: "This public survey group recruitment via self-selection will aim at reaching currently affected individuals who have not necessarily presented to outpatient or inpatient care, or have not been diagnosed with PCC by a GP or medical specialist. Despite the bias associated with self-reporting the presence of PCC symptoms, this potentially vulnerable group is considered separately. Individual additional questions are asked to validate the self-report."

4. The claims data should be clarified, as readers may not be familiar with the system

We have reworded the part on claims data in the abstract and added a sentence in the methods section to make it clearer.

Line 44-46: "(1) 21,000 adult individuals with a PCC diagnosis (ICD10 U09.9!) in their statutory health insurance claims data in 2022 ("AOK survey")"

Line 242-248: "The claims data of the 27,275 adult individuals with a PCC diagnosis in 2022 who are insured by AOK Lower Saxony (AOK claims data group) will serve as a secondary data source. The routine data of the statutory health insurers contain, in addition to the insured persons' sociodemographic data, billing data from reimbursed healthcare services providing an overview of the services utilised by patients from the time of diagnosis, with respect to outpatient care, medication, therapeutics, inpatient care and rehabilitation, and periods of sick leave incurred."

5. Provide the survey questionnaire contents to be used for GPs.

Unfortunately, we cannot provide the content of the survey for the general practitioners at this stage of the project, as it is still being identified based on the results of the focus groups.

6. Provide justification how the survey conducted in Lower Saxony can be generalizable to Germany, or provide as limitation in the discussion section.

Thank you for this advice. We have added additional information to the discussion.

Line 408-414: "Lower Saxony in general comprises nearly 10 % of the German population and is made up of rural, urban and metropolitan regions. In terms of age structure and unemployment rate, Lower Saxony is very close to the national average. The proportion of foreigners, the proportion of the employed persons with academic qualifications and the net income per capita are slightly lower than the German average [52]. However, due to the rather

small differences, the results of this study are expected to be transferable to the whole of Germany."

7. The expected duration of the survey is not included and should be provided.

We added this information in the methods section.

Line 230 (patients): "Answering the survey is estimated to take about 30 minutes."

Line 288-289 (informal caregivers): "Answering the survey is estimated to take about 15 minutes."
